# Genomic Sequencing of Two Coffee-Infecting Strains of *Xylella fastidiosa* Isolated from Brazil

**DOI:** 10.1128/genomeA.01190-13

**Published:** 2014-01-16

**Authors:** Valquíria C. Alencar, Deibs Barbosa, Daiene S. Santos, Ana Cláudia F. Oliveira, Regina C. de Oliveira, Luiz R. Nunes

**Affiliations:** Núcleo Integrado de Biotecnologia, Universidade de Mogi das Cruzes (UMC), Mogi das Cruzes, São Paulo, Brazila; Centro de Ciências Naturais e Humanas, Universidade Federal do ABC (UFABC), Santo André, São Paulo, Brazilb

## Abstract

Here, we describe the draft genome sequences of two *Xylella fastidiosa* strains: *Xf*6c and *Xf*32, which have been obtained from infected coffee plants in Brazil, and are associated with the disease known as coffee leaf scorch (CLS).

## GENOME ANNOUNCEMENT

*Xylella fastidiosa* is a Gram-negative bacillus that has been implicated in the development of citrus variegated chlorosis (CVC) in orange trees and Pierce’s disease (PD) in vineyards. *X. fastidiosa* strains have also been found to infect other important crops, such as plums, mulberries, pears, oranges, grapes, almond trees, and ornamental plants (1), which has stimulated comparative genomic studies involving different disease-associated *X. fastidiosa* isolates (2). Coffee-infecting *X. fastidiosa* strains are believed to be responsible for another plant disease in South America known as coffee leaf scorch (CLS), which has the potential to cause severe economic losses. CLS symptoms include drying of infected branches, shortening of internode regions, decreased fruit size, chlorosis, and early senescence of leaves, affecting overall plant productivity (3). The disease was originally described in 1997 in the states of São Paulo and Minas Gerais, Brazil (4), and more recently, it has been identified in other important coffee-growing regions of the country, throughout the states of Espírito Santo, Paraná, Bahia, and Goiás (5).

In this paper, we report the assembly and preliminary analyses of the draft genomes for two CLS-associated *X. fastidiosa* strains, named *Xf*6c and *Xf*32, which have been obtained from infected coffee plants in the state of São Paulo. DNA samples from *Xf*6c and *Xf*32 were used to construct shotgun libraries with the aid of the GS DNA library preparation kit (Life Sciences/Roche). Mate-pair libraries were also constructed by randomly shearing genomic DNA into 8-kb average fragments using a Covaris S-series system (Woburn, MA). Whole-genome sequencing was performed in a Genome Sequencer FLX 454 (Life Sciences/Roche) using reagents from the GS FLX reagents (Roche Diagnostics), according to the manufacturer’s protocols and instructions (6). Sequence analysis and assembly were performed with the softwares GS FLX version 2.6 and GS *de novo* assembler version 2.6 (Roche Diagnostics).

The draft genomes of *Xf*6c and *Xf*32 have approximately 2.56 and 2.6 Mbp, respectively, which are likely to correspond to >95% of the full genomic sequences for these two bacteria, since the genomes of other *X. fastidiosa* strains display sizes ranging from 2.39 to 2.73 Mbp (2). Both draft genomes were annotated through submission to the NCBI Prokaryotic Genome Automatic Annotation Pipeline (PGAAP), resulting in the identification of 2,506 open reading frames (ORFs) in the genome of *Xf*6c and 2,477 ORFs in the genome of *Xf*32. We were able to verify that the two CLS-related strains share a total of 1,695 ORFs, including genes required for the maintenance of general metabolism and replication. Comparative analyses indicate the existence of variations in genes that produce toxins (which may help them to compete with endophytes present in different hosts), as well as surface factors (such as fimbrial adhesins and lipopolysaccharides [LPSs]), which are likely to be involved with recognition of specific host factors, which may influence host specificity, infectivity, and/or development of virulence capacity.

### Nucleotide sequence accession numbers.

The whole-genome shotgun projects have been deposited at DDBJ/EMBL/GenBank under the accession no. AWYH00000000 and AXBS00000000 for strains *Xf*32 and *Xf*6c, respectively.
